# Restoration of contact inhibition in human glioblastoma cell lines after MIF knockdown

**DOI:** 10.1186/1471-2407-9-464

**Published:** 2009-12-28

**Authors:** Jörg Schrader, Oliver Deuster, Birgit Rinn, Martina Schulz, Andreas Kautz, Richard Dodel, Bernhard Meyer, Yousef Al-Abed, Karthikeyan Balakrishnan, Jens P Reese, Michael Bacher

**Affiliations:** 1 Institute of Immunology, University of Marburg, Marburg, Germany; 2 I. Medical Department, University Hospital Hamburg-Eppendorf, Hamburg, Germany; 3 Department of Neurology, University of Marburg, Marburg, Germany; 4 Department of Neurosurgery, Technical University Munich, Germany; 5 Laboratory of Medicinal Chemistry, North Shore Long Island Jewish Research Institute, Manhasset, New York 11030, USA

## Abstract

**Background:**

Studies of the role of the cytokine macrophage-migration-inhibitory-factor (MIF) in malignant tumors have revealed its stimulating influence on cell-cycle progression, angiogenesis and anti-apoptosis.

**Results:**

Here we show that *in vitro *targeting MIF in cultures of human malignant glioblastoma cells by either antisense plasmid introduction or anti-MIF antibody treatment reduced the growth rates of tumor cells. Of note is the marked decrease of proliferation under confluent and over-confluent conditions, implying a role of MIF in overcoming contact inhibition. Several proteins involved in contact inhibition including p27, p21, p53 and CEBPalpha are upregulated in the MIF antisense clones indicating a restoration of contact inhibition in the tumor cells. Correspondingly, we observed a marked increase in MIF mRNA and protein content under higher cell densities in LN18 cells. Furthermore, we showed the relevance of the enzymatic active site of MIF for the proliferation of glioblastoma cells by using the MIF-tautomerase inhibitor ISO-1.

**Conclusion:**

Our study adds another puzzle stone to the role of MIF in tumor growth and progression by showing the importance of MIF for overcoming contact inhibition.

## Background

The cytokine macrophage migration inhibitory factor (MIF) has long been known as a modulator of the immune response towards various infectious agents [1-4] Over the last years, its role in other disease-related processes, in particular neoplastic disorders, has been elucidated [5]. MIF is expressed in various malignant tumors, comprising ectodermal [6], mesenchymal [7,8] and endodermal cell types [9,10]. MIF functions in multiple ways to boost tumor growth by promoting angiogenesis [11-13], stimulating cell cycle progression [8,10,14], inhibiting apoptosis [15,16] and preventing NK cell lysis [6]. MIF expression in tumor cell lines is regulated by growth factors [10] and cell stress [7,17]. Suppression of MIF function by anti-MIF antibody treatment and MIF-antisense transfection alters the proliferate state of tumor cells *in vivo *and *in vitro *[10,12,13]. Additionally, MIF has been identified as a promoter of carcinogenesis in an intestinal tumor model [18]. Recently, a MIF receptor complex consisting of the invariant chain of the MHC complex CD74 and the hyaluronate receptor CD44 has been identified [19]. The CD44 protein has also been implicated in mediating contact inhibition in various cell types [20].

The expression of MIF has been described in tumours of the central nervous system and the potential role for tumour developement and progression in the brain has been recently reviewed [21]. In particular a strong increase of MIF expression in human glioblastoma multiforme has been reported by several investigators [7,22,23]. Glioblastoma multiforme belongs to the most malignant tumors known in men [24]. They infiltrate and displace normal brain tissue and therefore must have invoked a potent mechanism to overcome classical contact inhibition.

The aim of the study was to find out whether MIF plays a role in these altered growth processes in gliomas and also to test whether it might be a promising target for cancer therapy. We therefore analyzed the growth of human glioma cell lines *in vitro *while targeting the MIF function in various ways. Special attention was drawn to proliferative responses under confluent and over-confluent cell culture conditions.

## Methods

### Cell Culture

Human glioma cell lines LN18 and LN229 were grown in Dulbecco's modified eagle medium (DMEM) (Gibco-Life Technologies, Eggenstein, Germany) supplemented with 5% fetal calf serum (FCS) (Seromed, Berlin, Germany), 1% penicillin/streptomycin (Serva, Heidelberg, Germany) and 1% L-glutamine (Gibco-Life Technologies, Eggenstein, Germany).

### Plasmid cloning

The entire human MIF cDNA was cloned in antisense orientation into the BamH1/EcoRV restriction sites of the pcDNA 3.1/Myc-His vector (Gibco-Life Technologies, Eggenstein, Germany).

### Transfection

The LN18 cells were transfected at semiconfluent cell density with the linearized antisense and control plasmid with the Lipofectamin reagent (Gibco-Life Technologies, Eggenstein, Germany). Stable transfectants were selected by adding 1 mg/ml G418 (Invitrogen, Leek, Netherlands) to the cultures. After 4 weeks, the remaining cells were plated out highly diluted and the emerging clones picked with a sterilized needle and further propagated. From the initially picked 24 antisense clones (Additional file 1), two of these (termed as1 and as2) were chosen for the experiments because of their high consistent MIF antisense production. Seven empty vector transfected clones were generated in a similar way and clone 5 (here after termed c1) was used as a control for all experiments with the antisenseMIF clones.

### RNA preparation and Northern blot analysis

Total RNA was prepared by the TRIzol method (Gibco-Life Technologies, Eggenstein, Germany). RNA samples (5 μg) were separated on 1% agarose gels. The RNA was blotted with 20× SSC (1× SSC is 0.15 M NaCl plus 0.015 M sodium citrate) onto a positively charged nylon membrane (Boehringer GmbH, Mannheim, Germany). After UV-cross-linking, hybridization was performed under continuous rotation in a hybridization oven (Biometra, Goettingen, Germany). The membranes were hybridized with digoxigenin (DIG)-labeled antisense riboprobes overnight under highly stringent conditions in 50% formamide at 68°C and finally washed in 0.1% SSC, 0.1% sodium dodecyl sulfate at the same temperature. Hybridized DIG-labeled riboprobes were visualized with the DIG nucleic acid detection kit (Boehringer) and CPD-Star chemiluminescence substrate (Tropix, Bedford, Mass.; distributed by Serva, Heidelberg, Germany). Equal loading of the RNA was confirmed by staining of 18S and 28S RNA wth methylene blue. Quantification of band intensities were done with ImageJ (NIH, Bethesda, USA) and normalized to 18S RNA. Results represent the mean of at least 4 independent experiments.

### Western blot

Protein samples were prepared by lyszing cells in 1% Triton containing proteinase and phosphatase inhibitors. Supernatans were concentrated 20-fold with 10 kDa Centricon filter columns (Millipore, Billerica, USA) prior to addition of sample buffer. Western blotting was performed by the NuPAGE electrophoresis system (Novex, San Diego, USA) using 4 to 12% *N*, *N*-methylenebisacrylamide-Tris gels. Proteins were transferred onto Optitran BA-S83 membranes (Schleicher & Schuell, Dassel, Germany). The antibodies used were polyclonal anti-human MIF (1:4000) rabbit immunoglobulin G (IgG) as described earlier [1], anti-p21 (1:1000), anti-p27 (1:1000), anti-C/EBPalpha (1:1000), anti-CD74 (1:500), anti-Erk2 (1:1000), anti-phospho-Erk (1:2000), anti-pan-Akt (1:1000), anti-beta-Actin (1:2000) (all from Santa Cruz, La Jolla, USA), anti-p53 (1:1000), anti-phospho-Akt (1:2000) (all from Cell Signaling, Danvers, USA), anti-CD44 (1:500)(BD Bioscience Pharmingen, Bedford, USA) and peroxidase-labeled goat anti-rabbit IgG and anti-mouse IgG diluted 1/2000 in 5% milk in TBS/0,05% Tween (Cell Signalling, Danvers, USA). The bands were visualized by an enhanced chemiluminescence detection system, as recommended by the manufacturer (SuperSignal ULTRA; Pierce, Rockford; USA). Quantification of band intensities were done with ImageJ (NIH, Bethesda, USA) and normalized to Actin if appropriate. Results represent the mean of at least 3 independent experiments.

### BrdU assay

The cells were plated out in the stated numbers (see figure legend) in 96-well plates (Costar Corporation, Cambridge, USA) and incubated for 24 h prior to the experiments. The cells were incubated for 6 h with fresh medium supplemented with either anti MIF-antibodies or the MIF-inhibitor ISO-1 and then for additional 2 h when the BrdU labeling reagent was added. For experiments with recombinant MIF cells were pre-treated with 10 and 50 ng/ml recombinant human MIF for 12 h before addition of BrdU labeling reagent for another 2 h. The assay was performed using the Cell proliferation ELISA, BrdU chemiluminescence (Boehringer, Mannheim, Germany) according to the manufacturers instructions. The final results were obtained by reading the chemiluminescence values (relative light units (rlu) with a Lumistar automated plate reader (bmg, Offenburg, Germany). Results are given as average of relative values of controls with SD of eight simultaneous experiments. Statistics were calculated using Mann-Whitney unpaired non-parametric test and a p-value < 0.05 was regarded significant. All experiments have at least been repeated three times with similar results.

### Amido-black-Assay

Over a period of several days, growth was assessed by staining the cells for intracellular protein content. The protein staining was accomplished by using the amido-black method [25]. In brief, the cells were plated out at 5000 cells/well on multiple 96-well plates and fed every other day. For experiments with addition of MIF to culture media, recombinant human MIF was added at 10 ng/ml and 50 ng/ml with every medium change. Every day, the cells in one plate were fixed and denatured with formaldehyde and stained with amido-black solution. After drying of the plate, the amido-black dye was eluted from protein bound with NaOH and the light absorption at 620 nm was recorded with an automated plate reader. Results are given as averages of relative values of controls with SD of eight simultaneous experiments. Statistics were calculated using Mann-Whitney unpaired non-parametric test and a p-value < 0.05 was regarded significant. The experiments have been repeated three times with similar results.

### MIF blocking antibody

The antibodies against human MIF were initially raised in rabbits, following a standard immunization protocol. Monoclonal antibodies were then produced from the fusion of myeloma cells and antibody-producing B-lymphocytes (Institute of Biotechnology Vilnius, Lithuania). Binding of the monoclonal antibodies to MIF was confirmed by specific ELISA The antibody was purified from ascites by sepharose columns using Kaptiv-M (Tecnogen, Piana di Monte Verna, Italy).

### Small compound MIF inhibitor

The synthetic MIF inhibitor, (S,R)-3-(4-Hydrophenyl)-4,5-dihydro-5-isoazole acetic acid methyl ester (ISO-1) has been recently shown to covalently bind to the D-dopachrome tautomerase activity site of MIF and inhibits several biological activities of MIF in vitro and in vivo [26]. The inhibitor ISO-1 (provided by Y. Al-Abed) was solubilized in DMSO at a concentration of 10 μg/μl (equivalent to 42 mM) and further diluted in PBS if required. For all experiments DMSO only treated cells served as a control.

### Recombinant human MIF

Human MIF cDNA was cloned into the pET-17b vector (Novagen, Madison, USA) and expressed in Escherichia coli BL-21 (DE3) strain after induction with 0.4 mM isopropyl -D-thiogalactopyranoside (IPTG) and purified from the soluble fraction of the cell lysate by two-step high-pressure liquid chromatography (HPLC): (i) size exclusion HPLC on Bio-Sil TSK 250 (Bio-Rad, Munich, Germany) and (ii) ion-exchange HPLC on Ultropac TSK CM-3SW (LKB/Pharmacia, Freiburg, Germany). Biological activity has been confirmed using proliferation assay in human fibroblasts (unpublished data) and calcium release assay [27].

### FACS analysis

Cells were harvested by trypsinization and blocked in human serum. Staining for CD44 and CD74 was performed using the following antibodies: anti-human CD44-PE (eBioscience, San Diego, USA), anti-human CD74-FITC (BD Bioscience, Bedford, USA) and corresponding isotype controls (eBioscience, San Diego, USA). Analysis was carried out on a BD LSR II (BD, Bedford, USA).

## Results

**Figure 1:**
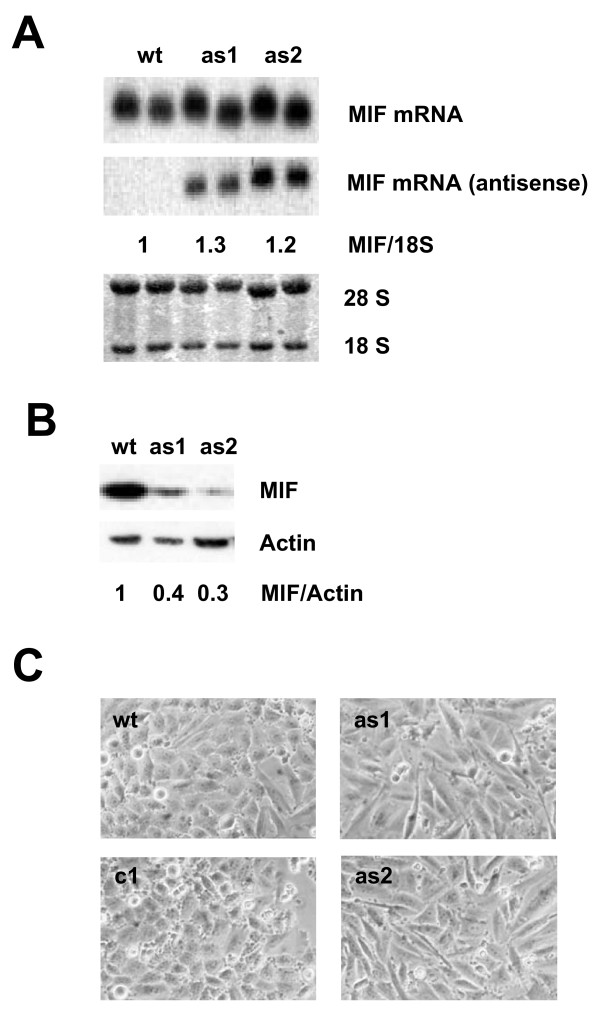
**MIF targeting by antisense transfection results in reduced MIF protein and altered morphology of glioma cells**. Human LN18 glioma cells transfected with an antisense-RNA MIF construct and corresponding antisense-expressing clones (as1 + as2) were selected (**A**). These cells showed a markedly reduced MIF protein expression (**B**) and altered cell morphology in culture compared to wildtype (wt) and empty vector transfected control cells (c1) (**C**). MIF expression ratios have been calculated from MIF expression relative to 18S (A) or Actin (B) in three independent experiments.

**Figure 2:**
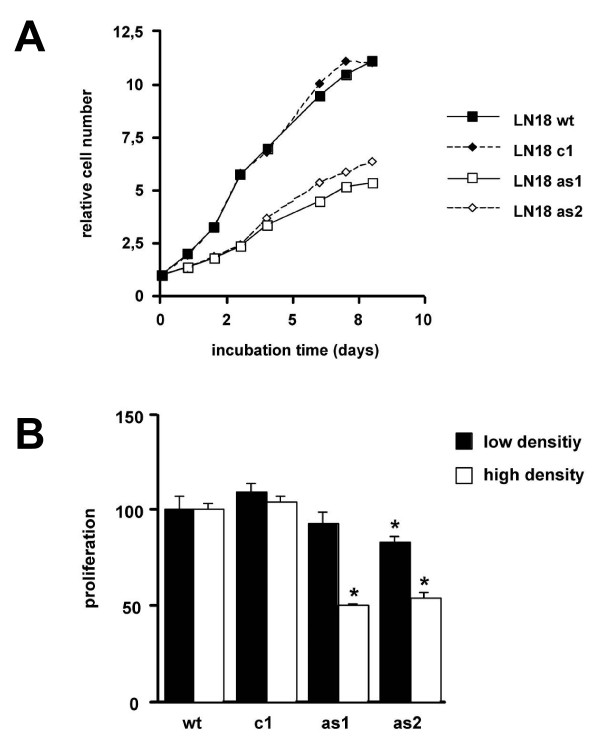
**MIF targeting by antisense transfection results in reduced proliferation and restoration of contact inhibition**. The generated LN18 MIF antisense-expressing clones (as1 + as2) and controls (wildtype wt, control - empty vector-transfected - clone c1) were plated on 96-well plates at the same cell number. Growth was measured the following days by amido-black assay. At day 8 the antisense clones as1 and as2 reached 48% (p 0.0043) and 57% (p 0.0007), respectively of the cell density of wildtype cells (**A**). DNA synthesis was significantly (* p < 0.005) compromised in clones at higher cell densities (**B**).

The generation of stable MIF-antisense-transfected clones is shown in Figure 1A. The MIF-antisense expression resulted in a reduction of MIF protein level by 60% in antisense clone as1 and 70% in the antisense clones as2 compared to wildtype (wt) cells (Figure 1B). It is of note that the antisense clones displayed an upregulated MIF-sense mRNA. In confluent cultures there is a marked difference in the morphologic appearance of wildtype and antisense clones. While the wt and the control vector-transfected cells (c1) display a more rounded and small cell shape in dense areas, the antisense clones rest in an ellipsoid and larger structure (Figure 1C). In semiconfluent cultures, the cells show nearly similar morphology **(data not shown)**. This morphologic difference correlated with an altered proliferation characteristic of the antisense clones. In a time kinetic growth analysis over eight days the antisense clones grew slower and reached only 48% (p 0.0043) and 57% (p 0.0007) respectively of the cell density of wildtype cells (Figure 2A). While having the same DNA synthesis rate at low densities, the rate was reduced in the as1 and as2 clones to 50% (p 0.0006) and 54% (p 0.0006), respectively at higher cell densities compared with wt and control cells (Figure 2B). Addition of recombinant human MIF to the wt and as clones resulted in a small increase in proliferation in the as clones in the short term with only marginal effects in wt and control cells, but did not lead to a sustained growth response in the long term (Additional file 2).

**Figure 3:**
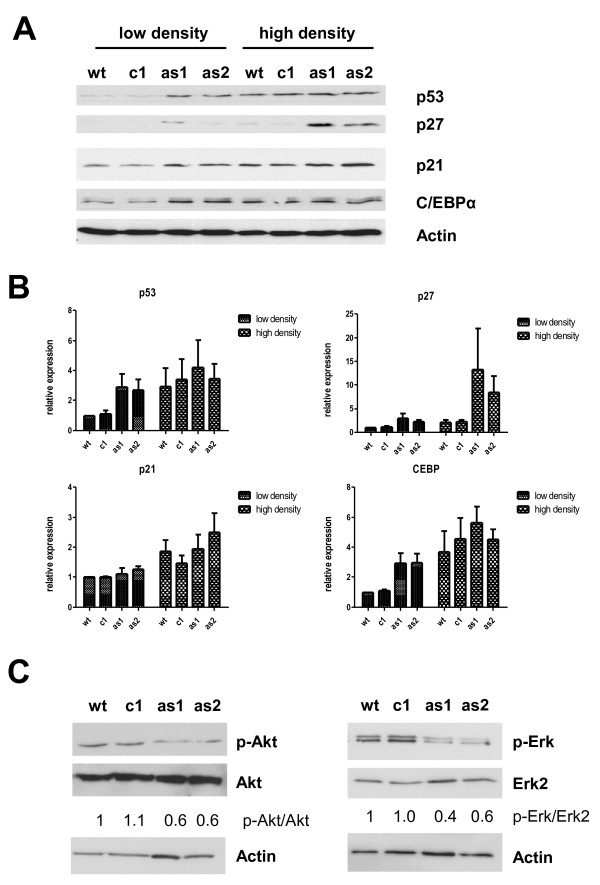
**MIF targeting results in upregulation of contact inhibition markers and reduced mitogenic signaling**. The antisense clones showed a restoration of contact inhibition indicated by increased expression of classical contact inhibition markers (p21, p27, p53 and C/EBPα) at lower cell densities (**A**). Densiometric quantification of the contact inhibitions markers relative to Actin of three independent experiments is shown in (**B**). Analysis of mitogenic signaling pathways revealed a marked reduction in basal Erk1/2 activity and a less pronounced reduction in Akt activity (**C**).

According to these functional data, we could show the increased expression of proteins associated with contact inhibition in the antisense-transfected LN18 cells at low plating density. The antisense clones showed an upregulated expression of p21, p27, p53 and CEBP/alpha at semi-confluent culture conditions compared to wt cells, (Figure 3A). When cells were plated at overconfluent cell density the wt and control cells also upregulated the above mentioned proteins, but there was still a higher expression of the cell cycle inhibitor p27 in the antisense clones. Since an influence of MIF on mitogenic signalling has been reported previously [11,28], we examined the activation of Akt and Erk in the antisense clones. We found a strong reduction of phospho-Erk and a modest reduction of phospho-Akt in the antisense clones compared to wildtype and control cells (Figure 3C).

**Figure 4:**
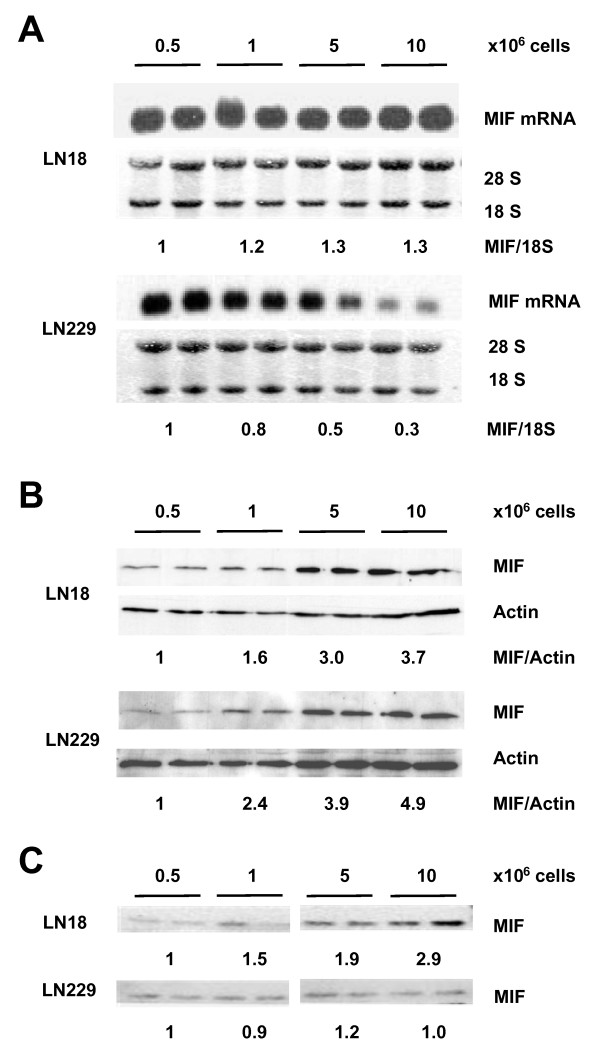
**Glioma cells exhibit an increasing MIF expression under higher cell densities**. The glioma cell lines LN18 and LN229 were plated at increasing cell densities. They display a much higher MIF protein content at high cell densities (**B**). The MIF mRNA expression was not affected by cell density in the LN18 cells, but was reduced in LN229 cells (**A**). Extracellular MIF levels remained constant (LN229) or increased (LN18) with cell density (**C**). MIF mRNA expression ratios relative to 18S RNA and MIF protein expression ratios relative to Actin were calculated from four independent experiments.

To further analyse the link between MIF and growth regulation we examined the expression of MIF at different cell densities. In addition to the LN18 cells we also studied another glioma cell line LN229. The LN229 cells were chosen because of morphological signs of contact inhibition at high cell densities which correlated with a reduced DNA synthesis rate at confluency (Additional file 3). In contrast, the LN18 cell cultures displayed overgrowth at higher cell densities and continued DNA synthesis at confluency. Analysis of the MIF mRNA expression under different cell densities of wildtype glioma cells, LN18 and LN229, revealed a marked difference between these cells. LN18 cells showed high MIF mRNA levels at all cell densities, while LN229 cells had reduced levels under confluent culture conditions (Figure 4A). Despite these differences in mRNA levels the intracellular MIF levels in both cell lines were increased at higher cell densities to a similar extend (3.7-fold and 4.9-fold for LN18 and LN229 cells, respectively) (Figure 4B). The extracellular MIF levels remained constant (LN229) or increased up tp 3-fold (LN18) (Figure 4C).

**Figure 5:**
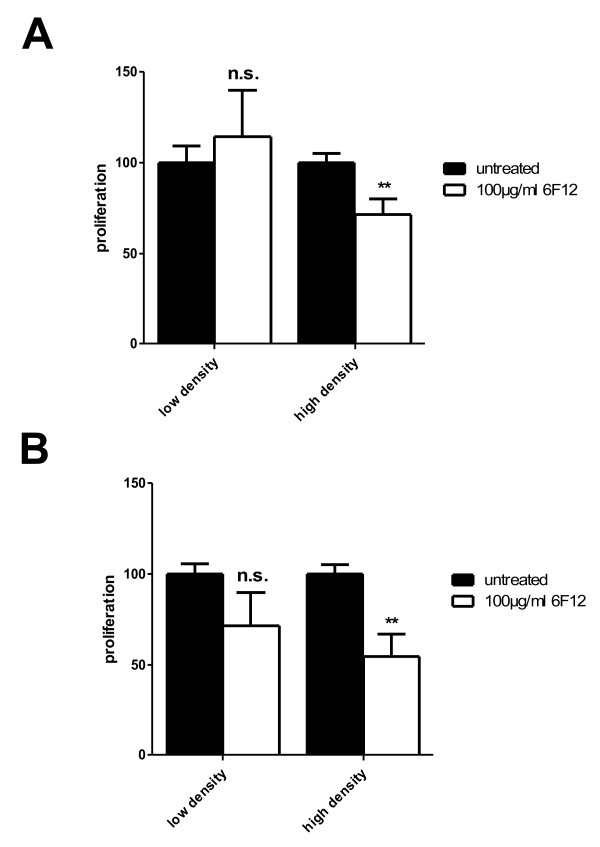
**Blocking MIF with a monoclonal antibody decreases proliferation of glioblastoma cell lines at high cell density**. The human glioblastoma cell lines LN18 (**A**) and LN229 (**B**) were incubated with a monoclonal MIF antibody (6F12) for 6 h. Measurement of DNA synthesis at low and high cell densities revealed a significant reduction of proliferation at high density (* p < 0.05, n.s. not significant).

**Figure 6:**
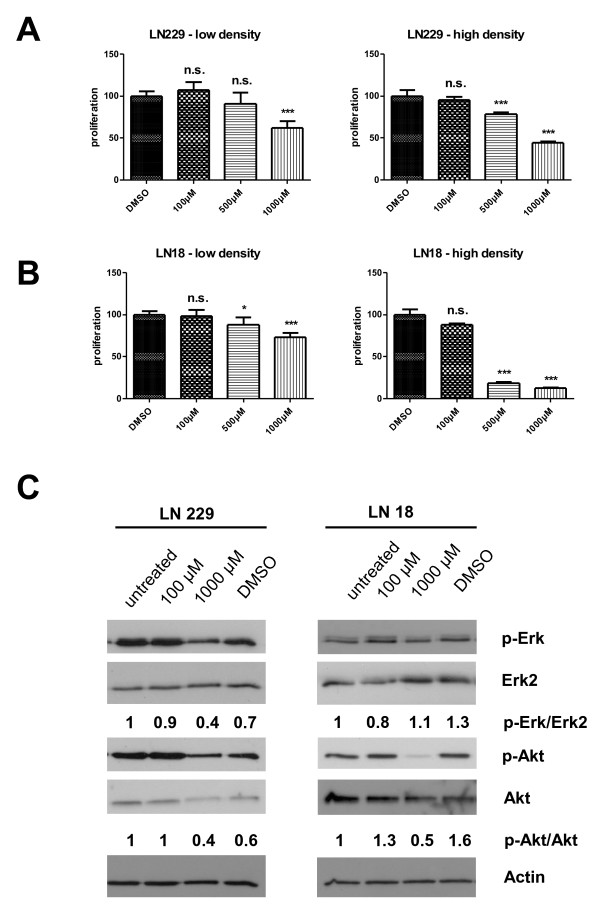
**Inhibiting MIFs intrinsic tautomerase activity by the small compound inhibitor ISO-1 reduced proliferation and mitogenic signalling in glioblastoma cells**. Small compound inhibitors can block the tautomerase activity of MIF. Incubating LN229 (**A**) and LN18 (**B**) cells with increasing concentrations of the inhibitor ISO-1 for 6 h (LN229) or 24 h (LN18) reduced cell proliferation. (* p < 0.05, *** p < 0.0005). Western Blot analysis of Akt and Erk1/2 phosphorylation showed a marked reduction in basal activity after ISO-1 treatment for 6 h in both cell lines (C). Akt and Erk phosphorylation ratios were calculated from two independent experiments.

When targeting the extracellular MIF protein by addition of monoclonal anti-MIF antibodies to the cell culture medium, there was only a modest growth reduction of LN18 cells under confluent culture conditions (30% of untreated cells, p 0.0286) with semiconfluent densities being non affected (Figure 5A). In contrast, LN229 cells showed a reduced growth rate at all cell densities, but still the maximal suppression (44%, p 0.0286) was seen under confluent culture conditions (Figure 5B). The use of the chemical inhibitor of the catalytic MIF activity, ISO-1, confirmed the results obtained with the antibody experiments. Similarly, the maximal growth reduction was elicited under the highest cell density (Figure 6A and 6B) without affecting cell viability **(data not shown)**. In the LN229 cells the maximal response was 56% reduction (p 0.0002) whereas in the LN18 cells the BrdU uptake could be suppressed even more (78%, p < 0.0001) compared to DMSO treated controls. The addition of ISO-1 to the cell cultures resulted in a decrease of the basal activity of the mitogenic signalling by Akt and Erk1/2 as shown by phospho-specific Western Blotting in both cell lines (Figure 6C).

**Figure 7:**
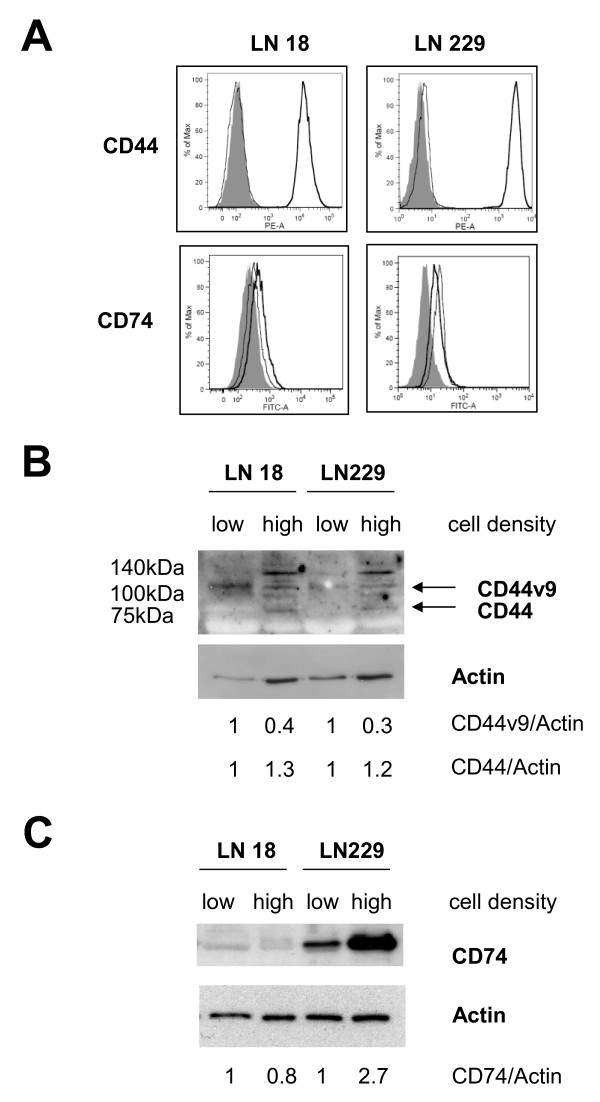
**LN18 and LN229 cells express the MIF receptor complex proteins CD44 and CD74**. FACS analysis revealed a strong expression of CD44 on both cell lines (A), which was confirmed by Western Blotting (B). Analysis of CD74 expression by FACS did not show a significant difference by flow cytometry (A), but strong expression of CD74 in the LN229 and weak expression in the LN18 cells could be detected by Western Blotting (C). Expression ratios for CD44 andCD74 relative to Actin have been calculated from three independent experiments. For FACS analysis filled area show autofluorescence of unstained cells and the thin graph represents the staining of the isotype control, whereas the signal of the PE labeled anti CD44 and FITC labeled anti CD74 is shown by the bold graph.

To confirm the presence of the MIF receptor proteins in the cells we performed FACS analysis and Western Blottting for CD44 and CD74. We detected a high expression of CD44 in both cell lines (Figure 7A) by FACS analysis, which was confirmed by Western Blotting (Figure 7B). Interestingly, at low density only high molecular weight isoform of CD44 could be detected, which represent the isofrom CD44v9. At higher cell density there is also expression of the normal 89 kDa isoform. Analysis of CD74 expression by flow cytometry gave only a weak signal, which was not higher than the corresponding isotype control (Figure 7A). But CD74 expression could be verified by Western blotting (Figure 7C). It is of note, that the protein levels were much higher in the LN229 cells and showed an upregulation by cell density in these cells. We did not find a regulation of either CD44 or CD74 upon MIF inhibition by the MIF inhibitor ISO-1 (Additional file 4).

## Discussion

In line with other studies on the role of MIF in tumor biology, our data show a profound influence of MIF targeting on the proliferative response in human glioma cells. Given the high grade of malignancy of glioblastomas [12], which normally implies many redundant growth promoting pathways, it seems puzzling that targeting a single protein gives responses with up to 60% growth reduction. This could indicate a pivotal role for MIF as a "second messenger" for other growth stimuli like EGF in an autocrine fashion. According to earlier studies there is a marked upregulation of MIF mRNA and protein after stimulation of colon derived cells with various growth factors [10]. This idea is also supported by the study of Mitchell and colleagues who found a sustained MAPK activity upon stimulation with MIF rather than a transient upregulation [28]. It is conceivable that MIF is a common permissive factor for other growth stimuli rather than possessing a direct stimulating activity. This model renders the MIF protein an interesting and promising target for further research in cancer therapy, because one could target a "common pathway" and not one of plenty alternatives.

One of the key events in tumor formation is the loss of contact inhibition resulting in an unrestricted growth of the cells despite overgrowth [29,30]. It has been shown previously that fibroblasts from MIF knockout mice reach lower cell densities than their wildtype counterparts [8]. Here we report for the first time a link between MIF and overriding contact inhibition in tumor cell lines. The expression of several proteins which mediate contact inhibition are modulated by MIF. Of note, the MIF antisense expressing cells show a much higher expression of classic cell cycle inhibitors like p21 and p27. In addition we found a reduction in basal state activity of the two mitogenic signalling pathways Erk and Akt, which might acount for the changes seen in cell cycle regulator expression. Similar obeservation have been made using siRNA targeting of MIF in prostate cancer [31] and ovarian cancer cells [32]. Furthermore, the antisense clones show a higher expression of the MIF sense mRNA, which might represent a mechanism to counterregulate the reduced MIF protein levels pointing to a tight feedback regulation of protein levels.

To our surprise, we were unable to rescue the phenotype of the as clones by adding recombinant human MIF to the cell cultures. Although the addition of recMIF to the as clones increased their proliferation in the short term, it did not fully restore normal growth nor growth kinetics in the longterm. Thus one might speculate, whether the effect of MIF in the LN18 cells is not only mediated by extracellular protein, but also by intracellular levels of MIF. Indeed, MIF has not only cell surface binding partners (e.g. CD74), but also intracellular binding partners (e.g. Jab1 and p53) which have well established roles in cell cycle regulation. Support for this hypothesis can be drawn from the fact, that the MIF inhibitor ISO-1 (which blocks extra- and intracellular MIF activity [33]) has a stronger effect on proliferation than MIF antibody treatment (blocks only extracellular MIF) in LN18 cells. In addition the LN18 cells show only a weak expression levels of the MIF receptor CD74, which has been correlated with the responsiveness of prostate cells to exogenous MIF [31]. A recent publication even reported a lack of CD74 expression in LN18 cells by PCR, confirming our findings [34]. Although the low CD74 expression in the LN18 cells most likely account for the unresponsiveness to exogeneous MIF, a different glioma cell line not expressing CD74 has been found to still be responsive to recombinant MIF - arguing for an alternative MIF receptor [34]. Another explanation for the reduced responsiveness of the as clones to exogeneous MIF might be possible post-translational modifications of endogenous MIF in opposition to recombinant MIF raised in E. coli, which might be important for the interaction with CD74 or alternative MIF receptors and for uptake of MIF into the cells.

Recently, the signal transduction of MIF has been linked to binding to CD74 and CD44. We could confirm the expression of both membrane proteins in our cell lines and found an increase in the expression with higher cell densities for in the LN229 cells. Interestingly, CD44 plays a key role in mediating contact inhibition on binding of hyaluronic acid [20]. Given the demonstrated binding of MIF to the CD74 and CD44 complex, an interference with the perception of contact inhibition signals by CD44 seems intriguing. Further research is needed to elucidate the exact mechanism involving the action of MIF under these circumstances.

It has long been debated whether the enzymatic tautoisomerase activity might be important for the biological function of MIF. Adding the specific MIF-tautomerase inhibitor ISO-1 to our cell cultures confirmed our results obtained with the antisense clones and antibody treatment. According to a recent study the enzymatic activity of MIF itself does not influence the biological functionality, but the conformation of the enzymatic domain is critical for the function of MIF [35]. The possible inhibitory activity of ISO-1 on MIF-promoted tumor suppression has recently been described in two in vivo models [31]. Here, we were able to extend these findings to a different tumour entity. In addition, we could show for the first time an effect of MIF inhibition by ISO-1 on mitogenic signalling by the MAPK/Erk and PI3K/Akt pathway. These data are in line with previous published reports targeting MIF by specific MIF neutralizing antibodies [11,28].

The regulation of the MIF expression by cell density gives more support to our hypothesis that MIF mediates the overcoming of contact inhibition. There was an increasee in MIF protein levels in both cell lines, despite constant (LN18) or even decreasing (LN229) mRNA levels with higher cell density. Since the extracellular MIF levels remain constant or are only disproportionately increased with increasing cell numbers (3-fold MIF upregulation, but 20-fold more cells), the increased MIF levels could be due to an accumulation of nonsecreted protein. A regulation of MIF protein levels by a post-translational mechanism affecting protein stability might be another mechanism accounting for the increase of intracellular MIF protein with cell densitiy despite similar or decreasing mRNA levels [36]. Another aspect of this finding is the MIF accumulation under cell stress, which also has been shown for various other agents and conditions inducing cell stress. Thus, the elevated MIF levels might help to protect the cells from induced cell death under stress by anti-apoptotic effects mediated by p53 [7,16]. Targeting MIF under these circumstances might alter the tumors' capability of withstanding radiation or chemotherapy, thereby increasing effectiveness and limiting side effects. Indeed targeting MIF by neutralizing antibody has recently been shown to enhance chemotherapy efficacy in breast cancer cells [37].

Our preliminary results from *in vitro *studies need to be followed by well-designed animal studies to further evaluate MIF function in cancer progression and in response to standard treatment. Our MIF antisense clones could be valuable tools for this purpose. In addition, the MIF-inhibitor ISO-1 used in this study could be a promising anticancer agent for further research.

## Conclusions

Our study provides new insight into the role of MIF in tumor growth and progression by showing the importance of MIF for overcoming contact inhibition.

We showed that *in vitro *targeting MIF in cultures of human malignant glioblastoma cells by either antisense plasmid introduction or anti-MIF antibody treatment reduced the growth rates of tumor cells. Of note was the marked decrease of proliferation under confluent and over-confluent conditions, implying a role of MIF in overcoming contact inhibition. Several proteins involved in contact inhibition including p27, p21, p53 and CEBPalpha were upregulated in the MIF antisense clones indicating a restoration of contact inhibition in the tumor cells. Furthermore, we showed the relevance of the enzymatic active site of MIF for the proliferation of glioblastoma cells by using the MIF-tautomerase inhibitor ISO-1

## Competing interests

The authors declare that they have no competing interests.

## Authors' contributions

JS and BR did all proliferation studies, transfections, and cell culture experiments. OD and AK performed FACS analysis and MS did Western Blotting. RD and BM provided intellectual contribution to the discussion. YAA provided the MIF inhibitor and contribution to the data analysis. KB and JPR did the RNA analysis. MB designed and planned the study.

## Pre-publication history

The pre-publication history for this paper can be accessed here:

http://www.biomedcentral.com/1471-2407/9/464/prepub

## Supplementary Material

Additional file 1 **Summary of all generated antisense MIF clones**. Northern Blot analysis for sense and antisense MIF of clones picked from LN18 cells stably transfected with either MIFasmRNA expressing plasmid or empty vector. Click here for file

Additional file 2 **Effect of rec. human MIF on proliferation of MIF antisense expressing LN18 clones**. Shortterm (BrdU-Assay) and longterm (Amidoblack-Assay) data on proliferation of MIF antisense clones and controls stimulated with various concentrations of recombinant human MIF. Click here for file

Additional file 3 **Comparison of growth characteristics of LN18 and LN229 cells under high confluency**. BrdU incorporation analysis of LN18 and LN229 cells plated at low and high cell density. Click here for file

Additional file 4 **CD44 and CD74 expression after ISO-1 treatment**. Flowcytometry analysis of CD44 and CD74 expression in LN18 and LN229 cells after treatment with the MIF inhibitor ISO-1. Click here for file
