# Supplementary material for: Restoration of contact inhibition in human glioblastoma cell lines after MIF knockdown
Source: BMC Cancer. 2009 Dec 28;9:464. doi: 10.1186/1471-2407-9-464 (PMC2810303; doi:10.1186/1471-2407-9-464)
Supplement: Additional file 1 — Summary of all generated antisense MIF clones. Northern Blot analysis for sense and antisense MIF of clones picked from LN18 cells stably transfected with either MIFasmRNA expressing plasmid or empty vector. [file 1471-2407-9-464-S1.PDF]

A

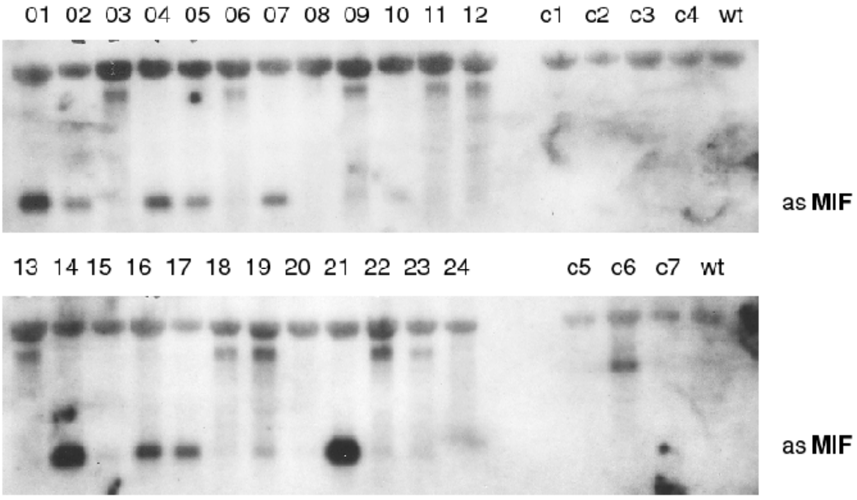

B

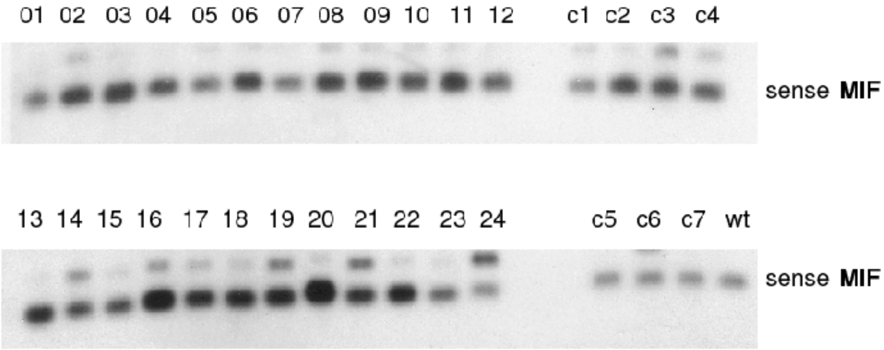

<u>Additional File 1:</u>

Northern Blot analysis of clones picked from LN18 cells stably transfected with either MIFantisense mRNA expressing plasmid (labelled 01-24) or empty vector (c1-c7). Blots were probed with sense MIF (A) and antisenseMIF (B).
