# Supplementary material for: Restoration of contact inhibition in human glioblastoma cell lines after MIF knockdown
Source: BMC Cancer. 2009 Dec 28;9:464. doi: 10.1186/1471-2407-9-464 (PMC2810303; doi:10.1186/1471-2407-9-464)
Supplement: Additional file 2 — Effect of rec. human MIF on proliferation of MIF antisense expressing LN18 clones. Shortterm (BrdU-Assay) and longterm (Amidoblack-Assay) data on proliferation of MIF antisense clones and controls stimulated with various concentrations of recombinant human MIF. [file 1471-2407-9-464-S2.PDF]

A

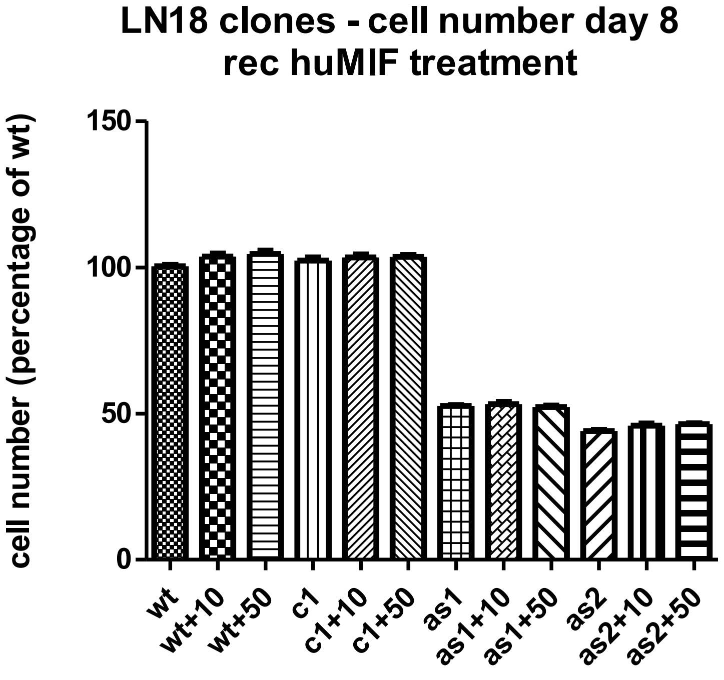

B

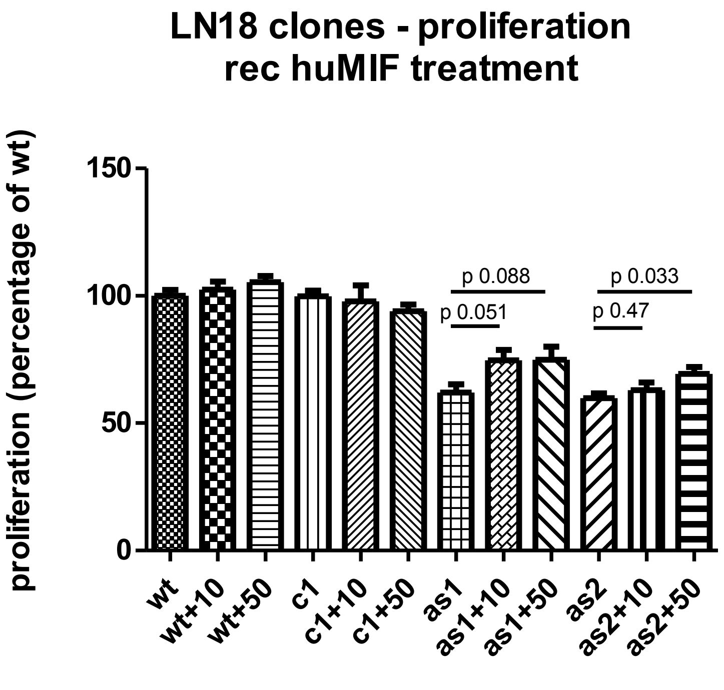

Additional File 2:

Ln18 cells and derived clones were plated at low density and cultured for 8 days in the presence of 0, 10 or 50ng/ml rec huMIF. Media was changed every second day with fresh supplementation of MIF. Cells were analyzed on day 8 by amidoblack-assay. Results are shown as mean of 8 replicates **(A)**.
LN18 cells were plated at full confluency and treated for 12h with rec huMIF 0, 10 or 50ng/ml before BrdU was added to measure DNA synthesis rate. Results are mean of 6 replicates **(B)**.
