# Supplementary material for: Restoration of contact inhibition in human glioblastoma cell lines after MIF knockdown
Source: BMC Cancer. 2009 Dec 28;9:464. doi: 10.1186/1471-2407-9-464 (PMC2810303; doi:10.1186/1471-2407-9-464)
Supplement: Additional file 3 — Comparison of growth characteristics of LN18 and LN229 cells under high confluency. BrdU incorporation analysis of LN18 and LN229 cells plated at low and high cell density. [file 1471-2407-9-464-S3.PDF]

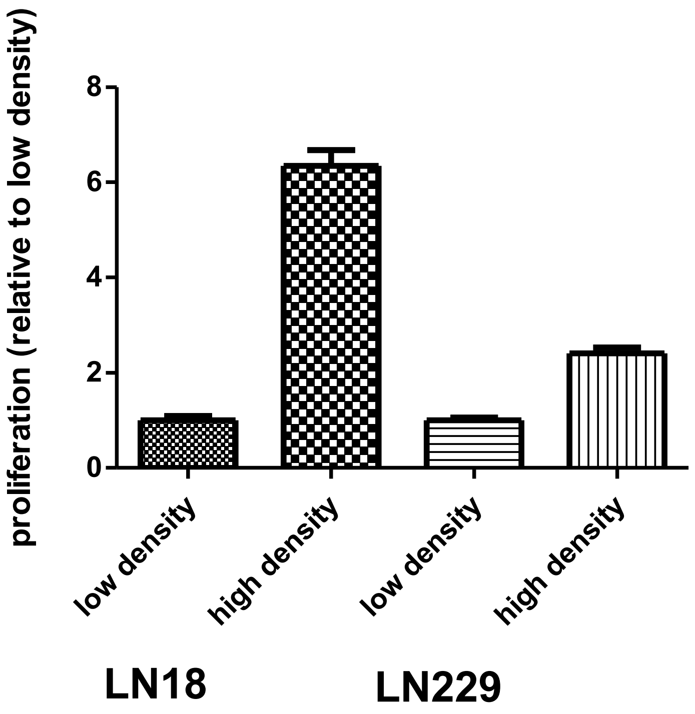

Additional File 3:

Proliferation rate of LN18 and LN229 cells under low and high cell density measured by BrdU incorporation assay.
