# Supplementary material for: Restoration of contact inhibition in human glioblastoma cell lines after MIF knockdown
Source: BMC Cancer. 2009 Dec 28;9:464. doi: 10.1186/1471-2407-9-464 (PMC2810303; doi:10.1186/1471-2407-9-464)
Supplement: Additional file 4 — CD44 and CD74 expression after ISO-1 treatment. Flowcytometry analysis of CD44 and CD74 expression in LN18 and LN229 cells after treatment with the MIF inhibitor ISO-1. [file 1471-2407-9-464-S4.PDF]

A

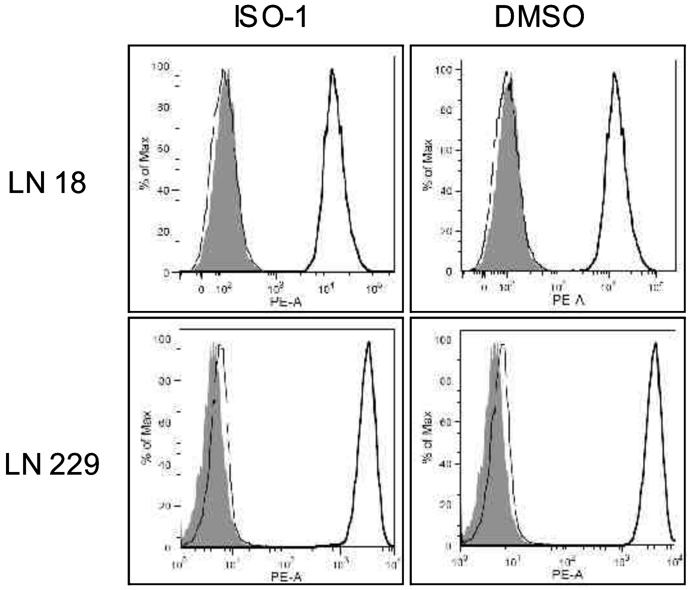

B

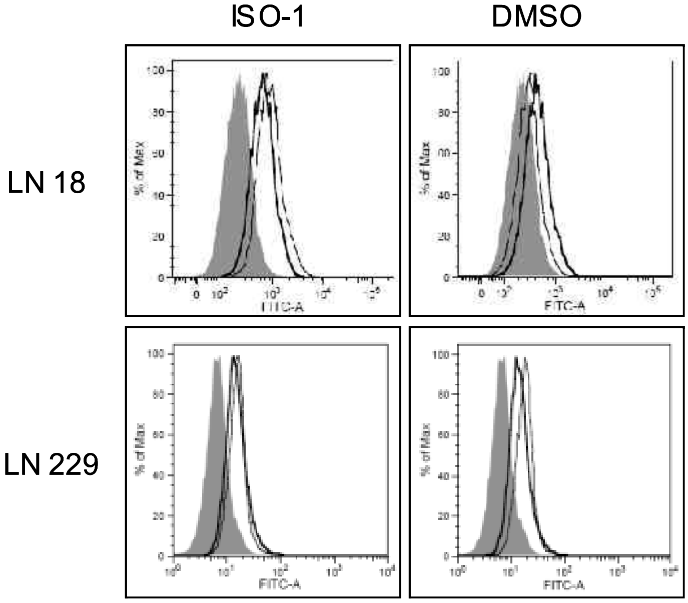

Additional File 4:

LN18 and LN229 cells treated with 200 µM ISO-1 and respective DMSO controls. Filled area shows autofluorescence of unstained cells. The thin graph represents the staining of the isotype control ab. The signal of the PE-labeled anti CD44 ab is shown by the bold graph **(A)**. The signal of FITC-labeled anti CD74 ab is shown by the bold graph **(B)**.
